# Nationwide assessment of fall risk and protective factors among older adults in Saudi Arabia

**DOI:** 10.1371/journal.pone.0332900

**Published:** 2025-09-24

**Authors:** Ahmad A. Alharbi, Abdulaziz A. Albalwi, Hamad S. Al Amer, Samia A. Alamrani, Hani F. Albalawi, Fawaz D. Alrashedi, Majed Y. Muthaffar, Mousa A. Albalwi, Yousef M. Alshehre, Hadeel R. Bakhsh, Maysoun N. Saleh

**Affiliations:** 1 Department of Health Rehabilitation Sciences, Faculty of Applied Medical Sciences, University of Tabuk, Tabuk, Saudi Arabia; 2 Department of Physical Therapy, King Fahad Hospital, Madinah, Saudi Arabia; 3 Department of Internal Medicine, Faculty of Medicine, University of Tabuk, Tabuk, Saudi Arabia; 4 Department of Rehabilitation Sciences, College of Health and Rehabilitation Sciences, Princess Nourah Bint Abdulrahman University, Riyadh, Saudi Arabia; 5 Department of Physiotherapy, School of Rehabilitation Sciences, The University of Jordan, Amman, Jordan; Al-Ahliyya Amman University, JORDAN

## Abstract

In Saudi Arabia, nearly half of older adults (49.9%) experience at least one fall annually. Despite its global and regional significance, research on fall risk factors in Saudi Arabia remains limited, with most studies concentrating on specific cities or subpopulations. This study investigated factors associated with falls among older adults across Saudi Arabia. In this cross-sectional study, 473 (58.8% female) older adults (58.4% aged 65–69 years) completed a questionnaire, where we collected data on demographic and health characteristics; additionally, participants completed the Arabic version of the Fall Risk Questionnaire (FRQ-AV). Both descriptive and inferential statistical methods, including logistic regression, were used to analyze the data using SPSS. Based on the FRQ-AV, 168 (35.5%) participants were identified as at risk of falling. After multivariable adjustment, the following significant risk factors were identified: age 70–74 (adjusted odds ratio [*a*OR]=2.52; 95% confidence interval [CI]: 1.35–4.68; *p* = 0.004) and ≥80 (*a*OR=4.85; 95% CI: 1.50–15.63; *p* = 0.008) years; coexisting diabetes and hypertension (*a*OR=3.59; 95% CI: 1.75–7.35; *p* < 0.0005); being somewhat (*a*OR=4.64; 95% CI: 1.78–12.12; *p* = 0.002), fairly (*a*OR=11.36; 95% CI: 4.04–31.91; *p* < 0.0005), or very afraid of falling (*a*OR=32.81; 95% CI: 10.88–98.99; *p* < 0.0005); and falling 1–2 (*a*OR=5.27; 95% CI: 2.80–9.89; *p* < 0.0005), 3–4 (*a*OR=13.06; 95% CI: 5.50–30.98; *p* < 0.0005), or five (*a*OR = 12.23; 95% CI: 2.88–51.83; *p* = 0.001) times in the past year. Protective factors included university education (OR=0.39; 95% CI: 0.22–0.68; *p* = 0.001) and engagement in physical activity (*a*OR=0.46; 95% CI: 0.26–0.81; *p* = 0.007). This study offers significant insights into fall factors and preventive strategies among older adults in Saudi Arabia. This study’s results can help in developing and implementing efficient fall prevention strategies nationwide.

## Introduction

Falls among older adults are a widespread and significant public health concern worldwide [1,2]. Globally, over a quarter of individuals aged ≥65 years experience at least one fall annually [1]. In Saudi Arabia, the prevalence is even more concerning, with up to 49.9% of older adults experiencing at least one fall yearly [3–5]. Additionally, recurrent falls—defined as two or more falls within 1 year—are a significant concern among older adults in Saudi Arabia [3,4]. More than half of older adults who experience a fall report having at least two falls annually. This highlights the severity and frequency of this dilemma in Saudi Arabia [3,4].

The consequences of falling among older adults can be severe and far-reaching, impacting physical health and the overall quality of life [6]. Falls are a leading cause of injury, frequently resulting in fractures, head trauma, or internal bleeding. These injuries may require prolonged hospitalization, surgery, and lengthy rehabilitation and can lead to long-term disability and early mortality [7]. Beyond the physical consequences, falling can have profound psychological effects [8]. Many older adults who experience a fall develop a fear of falling, resulting in physical inactivity, social detachment, and a decline in overall function. This fear of falling can create a vicious cycle, where inactivity leads to additional deterioration of muscular strength and balance, thereby increasing the risk of subsequent falls [8].

The serious negative consequences of falls highlight the critical need to identify and address factors associated with falls among older adults. Addressing these factors is crucial for developing comprehensive fall prevention strategies to enhance the safety and quality of life of older adults. Numerous studies have explored factors associated with falls in older individuals [4,5,9–12]. These studies have highlighted several factors, including age-related declines in muscle strength, balance, and vision, as well as the presence of chronic health conditions [4,5,9–12]. Conversely, most of these studies were conducted in Western countries. However, Saudi Arabia presents a unique context where distinct living conditions, cultural factors, and healthcare systems are rapidly evolving. Therefore, there is a clear need for localized research to understand the specific factors that contribute to falls among older adults in Saudi Arabia.

Few studies have been conducted in Saudi Arabia that examined fall risk factors among older adults [3–5,13,14]. However, while these studies provide valuable information, they focused on specific cities, including Riyadh [3], Unaizah [4], and Jeddah [13] or specific populations such as adult mothers [14]. Currently, no comprehensive nationwide study using a validated tool has been conducted to describe the factors associated with falls among older adults. Therefore, this study aimed to identify the factors associated with falls among older adults in Saudi Arabia. This knowledge will help develop targeted, culturally appropriate fall prevention strategies to address the needs of this growing population.

## Materials and methods

### Study design and participants

This cross-sectional analytical study employed convenience sampling and was conducted among older individuals attending community centers and healthcare providers across five regions in Saudi Arabia. However, a chi-square test indicated that the sample was not equally distributed across regions (χ² = 81.45, *p* < 0.001). This was due to logistical challenges during data collection. The sample size was determined using prevalence-based calculations, considering existing data on fall prevalence among older adults in Saudi Arabia [3]. A previous study has reported a 1-year fall prevalence of approximately 50% among the older population in Riyadh [3]. The required sample size was calculated using the standard formula for cross-sectional studies as follows:


$$\text{n}=\;{\text{Z}}^{\text{\textbackslash{}textrm\{2}}\}\text{P }(\text{\textbackslash{}textrm\{1}\}-\text{P})\;/\;{\text{d}}^{\text{\textbackslash{}textrm\{2}}\}$$

(where n: required sample size; Z: Z-score [1.96, 95% confidence level]; P: estimated prevalence of fall risk [assumed to be 50% or 0.50 based on prior studies]; d: margin of error [5% or 0.05]). Using these parameters, the minimum required sample size was calculated as 384 participants. To account for potential non-responses or incomplete data, we applied a 20% adjustment [15], resulting in a final target sample size of 461 participants.

Eligible participants were those aged ≥65 years, able to read and understand Arabic independently and capable of walking independently. Individuals with cardiovascular disease, uncontrolled hypertension (HTN), and/or orthopedic or neurological conditions disturbing their walking and balance were excluded.

### Ethics

The Institutional Review Board at the University of Tabuk approved the study (UT-249-95-2023). This study was conducted following the principles of the Declaration of Helsinki. All recruited participants were informed about the purpose of the study and provided with a written informed consent form before data collection.

### Procedure

Eight research assistants, primarily licensed physical therapists, participated in a 2-h training session designed to clarify the study methods before the initiation of data collection. The research assistants interviewed participants to complete the survey. Overall, 473 older adults were approached between February 1, 2023, and August 28, 2024, and recruited through community centers and primary healthcare clinics. Participants who met the study criteria were invited, and the research assistants discussed the informed consent with them. If they agreed to participate, the research assistants interviewed them to complete a survey.

### Measures

A survey comprising two sections was conducted to assess the factors associated with the risk of falls: the first section contained basic questions on sociodemographic characteristics of participants and health-related variables, including age, sex, height in meter (m), weight in kilograms (kg), body mass index (BMI, kg/m^2^; derived from height and weight and was calculated by dividing weight in kg by height in m^2^) engagement in physical activity, presence of chronic health condition, and history of falls. The second section included the Arabic version of the Fall Risk Questionnaire (FRQ-AV).

#### FRQ-AV.

The FRQ-AV is a screening questionnaire comprising 12 items designed to evaluate personal fall risk awareness in older populations. It can be easily completed by either older adults or healthcare professionals [16,17]. Each item on the checklist includes an explanatory note detailing its importance. Numerical values are assigned to “yes” or “no” responses, with individual items scored either 0–1 or 0–2, depending on the specific assessment. The FRQ has demonstrated a sensitivity of 73–80% in identifying fallers and predicting future falls among community-dwelling older adults in the United States [18]. The questionnaire was translated and validated in various languages, including Arabic [19,20]. The FRQ-AV’s threshold score is 7.5 points, above which an individual is considered to have an elevated fall risk associated with 73.7% sensitivity and 73.6% specificity [20].

### Data analysis

The data were summarized using descriptive statistics. For categorical variables, frequencies and percentages were calculated, whereas for continuous variables, median and interquartile range (IQR) were used due to non-normal distribution. Participants were first categorized based on their scores on the FRQ-AV as at risk of falls (FRQ score ≥ 7.5) and not at risk (FRQ score < 7.5) [20]. Next, univariable logistic regression analysis was conducted to calculate the odds ratio (OR) and 95% confidence intervals (CI) for each potential predictor. The univariable model was used to determine the factors that exhibited an effect without accounting for other variables [21]. Forward stepwise logistic regression analysis was also performed to compute the adjusted OR (*a*OR) [22], with a *p*-value threshold of 0.05 and 0.10 for variable entry and removal, respectively [23]. All variables potentially linked to fall risk were incorporated into the final multivariable logistic model. The significance of the association between risk factors and falls was determined at the 5% level (*p* < 0.05 was deemed significant) [21]. All statistical analyses were performed using IBM SPSS Statistics for Windows, version 26 (IBM Corp., Armonk, N.Y., USA).

## Results

### Participants

**Fig 1:**
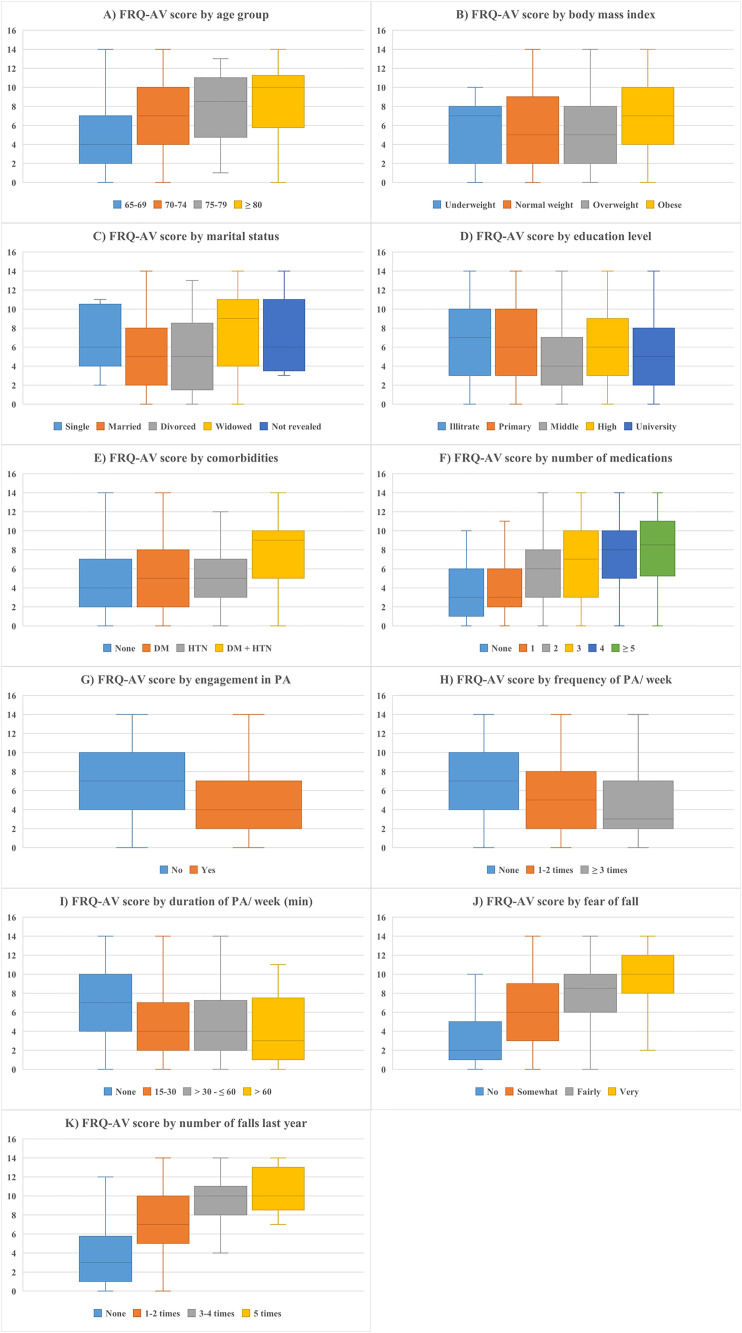
Distribution of FRQ-AV scores across levels of fall risk factors among study participants. Each boxplot displays the distribution of FRQ-AV scores across categories of independent variables. The central line in each box indicates the median, the box represents the interquartile range, and the lower and upper whiskers mark the minimum and maximum values, respectively. FRQ-AV: Arabic version of the Fall Risk Questionnaire; DM, diabetes mellitus; HTN, hypertension; PA, physical activity; min, minutes.

Overall, 473 participants were included in this study. Their demographic and health characteristics are presented in Tables 1 and 2, respectively. Most respondents were 65–69 years of age (58.4%) and married (78%). Additionally, 39% had a high school education or above, 34% had less than a high school education, and the remainder had no formal education. Moreover, 34.9% reported being somewhat “afraid” of falling. Regarding fall incidence, 50% had no falls during the previous year, while the other 50% reported one to five falls. The median (IQR) FRQ-AV score of the respondents was 6.0 (6.0). The variation in FRQ-AV scores based on age, BMI, physical activity, fear of falling, and other factors is visually summarized in Fig 1.

**Table 1:** Sociodemographic characteristics of the study participants (n = 473).

Characteristics		n	%
**Sex**	Men	195	41.2
	Women	278	58.8
**Age (years)**	65–69	276	58.4
	70–74	129	27.3
	75–79	42	8.9
	≥ 80	26	5.5
**Marital status**	Single	5	1.1
	Married	369	78.0
	Divorced	9	1.9
	Widowed	85	18.0
	Not revealed	5	1.1
**Geographical region in Saudi Arabia**	Southern	98	20.7
	Central	119	25.2
	Eastern	23	4.9
	Western	94	19.9
	Northern	139	29.4
**Education level**	No formal education	127	26.8
	Primary	98	20.7
	Middle school	63	13.3
	High school	79	16.7
	University	106	22.4
**Employment sector**	Education	77	16.3
	Administration	40	8.5
	Health	18	3.8
	Military	74	15.6
	Business trading	28	5.9
	Unemployed	236	49.9

**Table 2:** Health characteristics and profile of study participants (n = 473).

Weight median (IQR)		75.0 kg (19.0)	
**Height median (IQR)**		1.62 m (0.13)	
**BMI median (IQR)**		27.78 kg/m² (7.0)	
**FRQ-AR score median (IQR)**		6.0 (6.0)	
**Characteristics**		n	%
**History of chronic disease**	None	144	30.4
	Diabetes mellitus	78	16.5
	Hypertension	101	21.4
	Diabetes mellitus and hypertension	150	31.7
**Number of medications**	None	106	22.4
	1	65	13.7
	2	84	17.8
	3	75	15.9
	4	55	11.6
	≥ 5	88	18.6
**Engagement in PA**	No	228	48.2
	Yes	245	51.8
**Frequency of PA/ week** ^*^	1–2 times	81	17.1
	≥ 3 times	164	34.7
Duration of PA/ week^*^ **(min.)**	15–30	134	28.3
	> 30– ≤ 60	70	14.8
	> 60	41	8.7
**Fear of falling**	Not at all	150	31.7
	Somewhat afraid	165	34.9
	Fairly afraid	86	18.2
	Very afraid	72	15.2
**Number of falls in the last year**	None	240	50.7
	1–2 times	148	31.3
	3–4 times	64	13.5
	5 times	21	4.4

FRQ, Fall Risk Questionnaire; IQR, interquartile range; kg, kilogram; m, meter; BMI, body mass index; PA, physical activity.

^a^n = 245.

### Univariable logistic regression analysis of fall risk factors

As presented in Table 3, the results of the univariable logistic regression analysis revealed that the probability of being at risk of fall was significantly increased as the age increased from 70 to 74 (OR = 3.33; 95% CI: 2.13–5.20), to 75–79 (OR = 4.09; 95% CI: 2.10–7.99), and to ≥80 (OR = 7.61; 3.16–18.32) years. Obese (OR = 1.82; 95% CI: 1.11–2.97) and widowed (OR = 3.32; 95% CI: 2.04–5.40) participants had a higher risk of falls than normal-weight and married participants, respectively.

**Table 3:** Independent factors associated with fall risk (univariable and multivariable logistic regression) (n = 473).

Characteristics		Risk of fall		Univariable		Multivariable	
		No	Yes	OR (95% CI)	*p*-value	*a*OR (95% CI)	*p*-value
**Sex**	Men	127	68	1	–	–	
	Women	178	100	1.05 (0.72–1.54)	0.806	–	
**Age (years)**	65–69	213	63	1	–	1	–
	70–74	65	64	3.33 (2.13–5.20)^*^	< 0.0005	2.52 (1.35–4.68)^*^	0.004
	75–79	19	23	4.09 (2.10–7.99)^*^	< 0.0005	1.93 (0.69–5.41)	0.212
	≥ 80	8	18	7.61 (3.16–18.32)^*^	< 0.0005	4.85 (1.50–15.63)^*^	0.008
**BMI**	Underweight	5	2	0.89 (0.16–4.78)	0.889	–	
	Normal weight	82	37	1	–	–	
	Overweight	124	52	0.93 (0.56–1.54)	0.776	–	
	Obese	94	77	1.82 (1.11–2.97)^*^	0.017	–	
**Marital status**	Single	3	2	1.55 (0.26–9.40)	0.634	–	
	Married	258	111	1	–	–	
	Divorced	6	3	1.16 (0.29–4.73)	0.834	–	
	Widowed	35	50	3.32 (2.04–5.40)^*^	< 0.0005	–	
	Not revealed	3	2	1.55 (0.26–9.40)	0.634	–	
**Education**	No formal education	66	61	1	–	–	
	Primary	61	37	0.66 (0.38–1.12)	0.124	–	
	Middle	48	15	0.34 (0.17–0.66)^*^	0.002	–	
	High	52	27	0.56 (0.31–1.00)	0.052	–	
	University	78	28	0.39 (0.22–0.68)^*^	0.001	–	
**History of chronic disease**	None	112	32	1	–	1	–
	DM	55	23	1.46 (0.78–2.74)	0.233	1.65 (0.71–3.85)	0.248
	HTN	77	24	1.09 (0.60–1.99)	0.778	0.91 (0.41–2.02)	0.815
	DM + HTN	61	89	5.11 (3.07–8.51)^*^	< 0.0005	3.59 (1.75–7.35)*	< 0.0005
**Number of medications**	None	89	17	1	–	–	
	1	55	10	0.95 (0.41–2.23)	0.909	–	
	2	59	25	2.22 (1.10–4.46)^*^	0.025	–	
	3	41	34	4.34 (2.18–8.65)^*^	< 0.0005	–	
	4	27	28	5.43 (2.59–11.39)^*^	< 0.0005	–	
	≥ 5	34	54	8.31 (4.24–16.30)^*^	< 0.0005	–	
**Engagement in PA**	No	115	113	1	–	1	–
	Yes	190	55	0.29 (0.20–0.44)^*^	< 0.0005	0.46 (0.26–0.81)*	0.007
**Frequency of PA/ week**	None	115	113	1	–	–	
	1–2 times	58	23	0.40 (0.23–0.70)^*^	0.001	–	
	≥ 3 times	132	32	0.25 (0.15–0.39)^*^	< 0.0005	–	
**Duration of PA/ week (min)**	None	115	113	1	–	–	
	15–30	106	28	0.27 (0.16–0.44)^*^	< 0.0005	–	
	> 30– ≤ 60	53	17	0.33 (0.18–0.60)^*^	< 0.0005	–	
	> 60	31	10	0.33 (0.15–0.70)^*^	0.004	–	
**Fear of falling**	Not at all	144	6	1	–	1	–
	Somewhat afraid	110	55	12.00 (4.99–28.89)^*^	< 0.0005	4.64 (1.78–12.12)^*^	0.002
	Fairly afraid	35	51	34.97 (13.89–88.02)^*^	< 0.0005	11.36 (4.04–31.91)^*^	< 0.0005
	Very afraid	16	56	84.00 (31.28–225.6)^*^	< 0.0005	32.81 (10.88–98.99)^*^	< 0.0005
**Number of falls last year**	None	212	28	1	–	1	–
	1–2 times	75	73	7.37 (4.43–12.26)^*^	< 0.0005	5.27 (2.80–9.89)^*^	< 0.0005
	3–4 times	15	49	24.73 (12.28–49.80)^*^	< 0.0005	13.06 (5.50–30.98)^*^	< 0.0005
	5 times	3	18	45.43 (12.58–164.1)^*^	< 0.0005	12.23 (2.88–51.83)^*^	0.001

OR, odds ratio; CI, confidence interval; *a*OR; adjusted odds ratio; BMI, body mass index; DM, diabetes mellitus; HTN, hypertension; PA, physical activity

* Significant at α = 0.05.

The results showed that neither diabetes mellitus (DM) nor HTN was a risk factor for falls. However, the odds of falling were five times higher for those with both conditions (OR = 5.11; 95% CI: 3.07–8.51). Furthermore, the likelihood of being at risk of fall was almost the same for those taking one medication and those who were not (OR = 0.95; 95% CI: 0.41–2.23). However, the likelihood significantly increased as the number of medications rose from 2 (OR = 2.22; 95% CI: 1.10–4.46), to 3 (OR = 4.34; 95% CI: 2.18–8.65), to 4 (OR = 5.43; 95% CI: 2.59–11.39), and to ≥5 (OR = 8.31; 95% CI: 4.24–16.30) medications.

The odds became greater as the expression of fear increased from “somewhat afraid” (OR = 12.00; 95% CI: 4.99–28.89), to “fairly afraid” (OR = 34.97; 95% CI: 13.89–88.02), and to “very afraid” (OR = 84.00; 95% CI: 31.28–225.6). Similarly, the probability of risk of fall became higher as the number of fall incidences increased to 1–2 (OR = 7.37; 95% CI: 4.43–12.26), 3–4 (OR = 24.73; 95% CI: 12.28–49.80), and 5 (OR = 45.43; 95% CI: 12.58–164.1) times.

Performing any physical activity was found to have protective value against falls. Those who reported performing physical activity were less likely to fall than those who did not (OR = 0.29; 95% CI: 0.20–0.44). The odds were significantly lower among those who reported performing these activities one or two times a week (OR = 0.40; 95% CI: 0.23–0.70) and three times or more per week (OR = 0.25; 95% CI: 0.15–0.39). Additionally, performing these physical activities for weekly durations of 15–30 min (OR = 0.27; 95% CI: 0.16–0.44), 30–60 min (OR = 0.33; 95% CI: 0.18–0.60), and >60 min (OR = 0.33; 95% CI: 0.15–0.70) were found to be protective factors against falls. Lastly, participants with middle education (OR = 0.34; 95% CI: 0.17–0.66) and university degrees (OR = 0.39; 95% CI: 0.22–0.68) had a lower fall probability than their illiterate counterparts.

### Forward stepwise logistic regression analysis of fall risk factors

The forward stepwise logistic regression analysis confirmed the effect of age, history of chronic conditions, fear of falling, and number of falls as risk factors for falls (Table 3). Age groups 70–74 years (*a*OR = 2.52; 95% CI: 1.35–4.68) and ≥80 years (*a*OR = 4.85; 95% CI: 1.50–15.63) were significantly associated with fall risk. Having DM and HTN increased the likelihood of falls by more than three times that of those with neither condition (*a*OR = 3.59; 95% CI: 1.75–7.35). Similar to the findings of the univariable analysis, the likelihood became higher as fear increased from “somewhat afraid” (*a*OR = 4.64; 95% CI:1.78–12.12), to “fairly afraid” (*a*OR = 11.36; 95% CI: 4.04–31.91), and to “very afraid” (*a*OR = 32.81; 95% CI: (10.88–98.99). Likewise, the odds increased as the number of fall incidences increased to 1–2 (*a*OR = 5.27; 95% CI: 2.80–9.89), 3–4 (*a*OR = 13.06; 95% CI: 5.50–30.98), and 5 (*a*OR = 12.23; 95% CI: 2.88–51.83) times.

These results also confirmed physical activity as a protective factor against falls. The probability of being at risk of fall decreased by almost 50% among those engaging in physical activities compared to those who were not (*a*OR = 0.46; 95% CI: 0.26–0.81).

## Discussion

Every year, a significant number of older adults in Saudi Arabia experience falls. Approximately 35.5% of the participants in this study were at risk of falls, which is consistent with the national and global prevalence rate [2,4,24]. This study identified several factors significantly associated with fall risk, including age, history of chronic diseases, number of medications, fear of falling, and number of falls. However, regular physical activity has been identified as a significant protective factor against falls.

The risk of falls increases with age, with individuals aged >70 years at a significantly higher risk and an even greater risk for those aged >80 years. Research has consistently shown a strong association between aging and an increased risk of falls [25]. This was mostly justified by age-related frailty, a condition that is highly prevalent among older adults [26] and is characterized by a decline in physical strength and balance, frequently leading to falls and related serious health complications [27,28].

The prefrailty stage is a reversible intermediate phase between health and frailty. This offers an opportunity for targeted interventions to reduce the risk of falls in this group [26]. Early identification of pre-frail individuals through screening is essential for the timely implementation of preventive strategies [29,30]. Various interventions exist; however, multicomponent physical exercise interventions—combining strength, resistance, balance, and gait training were the most effective in preventing the onset of frailty [31]. These approaches have shown greater effectiveness than interventions that are based on dietary changes, cognitive training, or environmental modifications [32]. We recommend screening programs for older adults to identify high-risk individuals. While training preventive programs are essential for all older adults, they are particularly crucial for those at a high risk of falling.

This study shows that neither DM nor HTN is a risk factor for falling. However, the coexistence of both conditions was a significant risk factor, with more than a threefold increase in the risk of falls (*a*OR = 3.59). Previous studies have recognized the association between a history of chronic diseases and the risk of falls [33,34]. Individuals with DM may experience complications, including peripheral neuropathy, vestibular dysfunction, cognitive impairment, musculoskeletal or neuromuscular problems in the lower limbs, dizziness, and hypoglycemia, specifically with insulin use [35]. All these factors contribute to impaired balance and an increased risk of falls. According to a meta-analysis, DM increases the risk of falls by 94% and 27% in patients treated with insulin and those not treated with insulin, respectively [36]. HTN has also been recognized as a risk factor for falls. In their meta-analysis, Hu et al. (2021) identified a significant association between frailty and increased risk of injurious falls in individuals with HTN [37]. Furthermore, as the number of medications increases, the risk of falls also increases. Evidence has shown that taking ≥4 medications is associated with an increased incidence of falls, recurrent falls, and injurious falls [38]. The combined effects of DM and HTN on mobility, balance, and cognition, along with the side effects of medications, may explain the increased risk of falls [33,34]. Therefore, prevention programs should prioritize the management of these conditions to effectively reduce the risk of falls. Although some evidence suggests that reducing the number of medications does not significantly reduce falls [39], multidisciplinary team interventions are still required. Healthcare providers should collaborate to assess the patient’s ultimate need for medications and determine the optimal number required. Moreover, rehabilitation specialists can assess fall risk factors and aid in referral to early intervention programs.

In our study, a history of previous falls and fear of falling emerged as strong predictors of fall risk. Individuals with a history of falls, especially those who had fallen three to four or more than five times, were found to have a significantly higher risk. Moreover, participants who reported a fear of falling were more likely to experience future falls than those without such fears. Evidence supports these findings, where Pena et al. (2019) showed that older adults with a fear of falling are up to 12 times more likely to fall [40]. Researchers have found an increased fracture risk in older adults who have experienced a fall [41]. These individuals frequently develop a fear of falling, which can lead to reduced physical activity and a poor quality of life [42]. This fear may produce a “fear-avoidant” behavior, where inactivity results in muscle weakness and balance impairment, increasing the risk of recurrent falls [43]. In severe cases, frequent falls and related health problems can lead to long-term disabilities, loss of independence, and mortality. These findings emphasize the psychological effects of falls and the need to address these fears through supportive therapies [44]. Awareness and prevention programs targeting individuals with a history of falls may effectively reduce their risk of recurrent falls [45,46]. Therefore, we strongly recommend establishing educational programs for older adults and their families on preventive measures to reduce the risk of falls. These educational programs should be implemented at rehabilitation and primary care centers to reach all older adults.

In this study, physical activity played a protective role in reducing the risk of falls. Regular physical activity was significantly associated with a lower risk of falls, confirming that even moderate regular exercise helps maintain strength and balance. This finding is consistent with that of previous reviews showing that long-term moderate-intensity exercise performed two to three times per week, combined with balance training, reduces fall risk and injury rates in older adults [47,48]. Resistance exercise training was the most cost-effective exercise intervention for fall prevention [49]. This highlights the importance of promoting physical activity among older adults by focusing on muscle strength and balance training.

This study identified certain factors that may increase the risk of falls among older adults in Saudi Arabia; however, these factors were not significant in the stepwise logistic regression analysis. Obesity has been shown in previous research to be associated with a greater risk of falling and greater functional disability after a fall [50]. Research suggests that older adults with obesity are 16% more likely to fall than non-obese individuals [51]. Several factors contribute to this risk, including poor muscle quality [52], higher foot load [53], impaired postural balance, and increased fear of falling [54]. Therefore, incorporating weight control measures into fall prevention strategies is crucial. Similarly, our study found that widowed individuals are more likely to fall than their married counterparts. Evidence further supports this, showing that unmarried individuals have nearly twice the risk of frailty compared to married people [54]. This could be attributed to a decrease in physical and social support, underscoring the importance of considering marital status as a significant risk factor for falls [55]. Engaging single individuals in support groups and social activities may provide them with greater support and encourage more physical activity, potentially reducing their risk of falls.

Furthermore, our results demonstrated that a high educational level could be a protective factor against falls, which is consistent with a recent review [56]. Our findings suggest that individuals with higher education levels are more familiar with and adopt fall prevention behaviors, including engaging in regular physical activity. These findings highlight the importance of developing targeted educational interventions for individuals with low literacy levels. Importantly, such interventions should also extend to caregivers who often play critical roles in supporting elderly individuals’ daily health-related activities. Educating caregivers can enhance their ability to implement and sustain fall prevention measures in the home setting. Previous studies have demonstrated that fall prevention education programs for caregivers can significantly improve their knowledge, reduce caregiver burden, and enhance fall prevention behaviors [57,58].

This study found no significant association between sex and the risk of falls among older adults. This contrasts with previous literature that has found gender-specific patterns in fall risk. For example, a rural North Carolina study reported that women aged 71–80 years fell more frequently than men and were more likely to notify others about falls, live alone, and report a greater fear of falling—all factors potentially increasing their fall risk [59]. Similarly, the Canadian Community Health Survey identified different fall-related risk factors for men and women, emphasizing the role of sex in fall risk profiles [60]. The lack of a significant sex association in our findings may reflect differences in social support structures, cultural norms regarding gender roles, or health behaviors in the Saudi context. It may also indicate that other variables, including chronic conditions, number of medications, and physical activity, have a stronger influence on fall risk than sex alone. These observations emphasize how crucial context-specific risk assessments are to creating successful fall prevention plans.

This study stands out as one of the first to employ a validated and reliable tool for assessing fall risk among older adults in Saudi Arabia. The tool used was recently translated and culturally adapted to Arabic, making it relevant and applicable to the local population [20]. The FRQ-AV cutoff score used in this study was based on the validation research, which showed strong predictive validity for identifying fall risk in older adults [20].

A key strength of this study is the inclusion of participants from all regions of Saudi Arabia, enhancing the representativeness of the findings. Furthermore, this study’s comprehensive approach to examining various predictors of falls provides valuable insights for developing targeted fall prevention strategies. Some limitations of this study include the use of a single question to assess fear of falling and two questions regarding physical activity, rather than validated questionnaires specifically designed for these purposes. Cognitive impairment screening for the participants in our study was not conducted. However, our approach produced significant results. Additionally, the cross-sectional design may limit the ability to establish a causal relationship between fall risk and other factors and is also subject to recall and selection bias. Although participants were recruited from all five geographical regions of Saudi Arabia, the sample distribution was uneven. Future research should aim to ensure balanced sampling across regions to enable more generalizable conclusions. Despite these limitations, our findings provide an important foundation for fall prevention programs in Saudi Arabia. Future studies could explore the influence of cultural and environmental factors, including housing conditions and family support. This will enhance the understanding of fall risk and aid in developing more effective and culturally tailored preventive strategies.

## Conclusions

This study highlights the urgent need for fall prevention among the older population in Saudi Arabia. Addressing manageable risk factors, such as physical inactivity and fear of falling, can significantly reduce the incidence of falls. Comprehensive workout regimens incorporating strength, balance training, and psychological support are essential. Standard health care for older adults should encompass fall risk assessments and preventive measures. By addressing these challenges, we can help older individuals maintain their independence and quality of life. Falls pose significant challenges to the health system and necessitate multisectoral collaboration, including policymakers, healthcare providers, and community organizations. This entails enhancing healthcare accessibility, increasing knowledge of fall risks, and fostering environments that are safe, accessible, supportive, and engaging as people age. Collectively, we can establish a more robust and supportive atmosphere for older individuals to age with dignity and autonomy. Rehabilitation services are core to fall risk prevention. Fitness and physical activity programs can be easily designed and implemented. Group exercises for older adults at community or rehabilitation centers may improve balance and postural control and increase social and psychological support for these individuals.

## Supporting information

S1 File This is the S1 File Dataset. (XLSX)

## Abbreviations

m: Meter
kg: Kilograms
BMI: Body mass index
FRQ-AV: Arabic version of the Fall Risk Questionnaire
IQR: Interquartile range
OR: Odds ratio
CI: Confidence intervals
*a*OR: Adjusted OR
DM: Diabetes mellitus
HTN: Hypertension
